# Hyperparameter tuning using Lévy flight and interactive crossover-based reptile search algorithm for eye movement event classification

**DOI:** 10.3389/fphys.2024.1366910

**Published:** 2024-05-15

**Authors:** V. Pradeep, Ananda Babu Jayachandra, S. S. Askar, Mohamed Abouhawwash

**Affiliations:** ^1^ Department of Information Science and Engineering, Alva’s Institute of Engineering and Technology, Mangaluru, India; ^2^ Department of Information Science and Engineering, Malnad College of Engineering, Hassan, India; ^3^ Department of Statistics and Operations Research, College of Science, King Saud University, Riyadh, Saudi Arabia; ^4^ Department of Mathematics, Faculty of Science, Mansoura University, Mansoura, Egypt

**Keywords:** accuracy, bidirectional long short-term memory, eye movement event classification, fuzzy data augmentation, F1-score, Lévy flight and interactive crossover, reptile search algorithm

## Abstract

**Introduction:** Eye movement is one of the cues used in human–machine interface technologies for predicting the intention of users. The developing application in eye movement event detection is the creation of assistive technologies for paralyzed patients. However, developing an effective classifier is one of the main issues in eye movement event detection.

**Methods:** In this paper, bidirectional long short-term memory (BILSTM) is proposed along with hyperparameter tuning for achieving effective eye movement event classification. The Lévy flight and interactive crossover-based reptile search algorithm (LICRSA) is used for optimizing the hyperparameters of BILSTM. The issues related to overfitting are avoided by using fuzzy data augmentation (FDA), and a deep neural network, namely, VGG-19, is used for extracting features from eye movements. Therefore, the optimization of hyperparameters using LICRSA enhances the classification of eye movement events using BILSTM.

**Results and Discussion:** The proposed BILSTM–LICRSA is evaluated by using accuracy, precision, sensitivity, F1-score, area under the receiver operating characteristic (AUROC) curve measure, and area under the precision–recall curve (AUPRC) measure for four datasets, namely, Lund2013, collected dataset, GazeBaseR, and UTMultiView. The gazeNet, human manual classification (HMC), and multi-source information-embedded approach (MSIEA) are used for comparison with the BILSTM–LICRSA. The F1-score of BILSTM–LICRSA for the GazeBaseR dataset is 98.99%, which is higher than that of the MSIEA.

## 1 Introduction

The human eye is considered a spontaneous way of understanding human communication and interaction, which is exploited for processing data according to the nearby environment, in response to the respective situation. The physiological capacities are highly constrained from creating movement in any of the limbs or the head, as a result of various diseases such as Parkinson’s, spinal cord injury, locked-in syndrome, muscular dystrophy, multiple sclerosis, complete paralysis, and arthritis. Hence, around 132 million disabled people require a wheelchair, and only 22% of them have access to one. Moreover, these disabled people cannot use a technically improved wheelchair. Therefore, an eye detection and tracking method is investigated for enhancing the interaction between humans and computers, and it will enhance the living standard of disabled people (Dahmani et al., 2020; Barz, and Sonntag, 2021; Koochaki, and Najafizadeh, 2021; Aunsri, and Rattarom, 2022). Brain activity triggers eye movements that are a response to visual stimuli or an intent to obtain information about the neighboring environment (Harezlak, and Kasprowski, 2020; Li et al., 2021; Vortmann, and Putze, 2021). Generally, eye movements are categorized into saccades and fixations, i.e., when the eye gaze moves from one position to another position and pauses at a certain position, respectively (Harezlak et al., 2019; Rahman et al., 2021; Yoo et al., 2021).

Eye tracking is the process of tracking and determining the movements of the eye and the focal point of the eye. The technology of eye tracking is used in various fields such as cognitive science, computer gaming, marketing, medicine, and psychology. Hence, eye tracking is extensively used in computer science applications by making use of the features of the eye for studying information processing tasks. In general, eye-tracking information is computed and acquired by using an eye-tracking sensor/camera. The acquired data offer many features and are useful in various classification tasks (Lim et al., 2022; Holmqvist et al., 2023). Eye tracking metrics are used for disclosing perceptions about the participant’s actions and mindset in different circumstances. Significant eye-tracking metrics are saccades, duration, pointing, fixation, and pupil diameter (Bitkina et al., 2021; Elmadjian et al., 2023). Eye movement classification is affected because eye-tracking data contain a huge amount of user data that are not required for all applications. For example, eye movement discovers characteristics such as bio-markers, identity, and gender (David-John et al., 2021).

In this research, eye movement event detection is performed using a deep learning classifier with hyperparameter tuning. Generally, hyperparameter tuning is used to choose the parameter values and obtain improved classification (Shankar et al., 2020). The major contributions of this research are given as follows:

• A BILSTM is used for classifying eye movement events to help disabled people. The BILSTM is used in this research because it considers both the past and upcoming data while classifying the given input.
• The LICRSA-based hyperparameter tuning has been proposed to optimize the following parameters: dropout, learning rate, L2 regularization, and max-epoch. The LICRSA is used because its Lévy flight approach helps discover out-of-local solutions. Next, interactive crossover increases the ability to search by acquiring the solution through optimal and remaining candidate solutions.

The remaining paper is organized as follows: Section 2 provides the details about existing eye-tracking applications. The proposed method is detailed in Section 4, whereas the outcomes of the proposed method are presented in Section 4. Finally, Section 5 offers the conclusion.

## 2 Related works

Li et al. (2020) implemented a machine learning-based automated approach to perform fatigue detection and classification for equipment operators. Toeplitz inverse covariance-based clustering (TICC) was used to obtain various mental fatigue levels and labeling based on the movement of the eye. Features of eye movement were acquired for various construction sites, and supervised learning classified the mental fatigue levels of the operator. TICC along with machine learning were used in various construction sites due to higher accuracy. An additional enhancement in accuracy was achieved only by using a large number of eye movement metrics related to mental fatigue.

Yang et al. (2023) developed an analysis for the attention patterns in depressed patients based on the region-of-interest (ROI) analysis. The established ROI recognition analysis was named ROI eventless clustering (REC) and did not need eye movement event discovery. For diverse attribute features, ROI clustering was operated with deflection elimination (RCDE) for supporting the discovery of depression. This RCDE also used noisy data for describing the attention patterns. Moreover, it was essential to use eye movement event because gaze features were vital while performing classification.

Mao et al., 2020 implemented disease classification according to the movements of the eye by using a deep neural network. Normalized pupil data such as size and location were offered as features of the eye movement. For each feature, long short-term memory (LSTM) was used for developing a weak classifier. The weights of each weak classifier were discovered using a self-learning method. Next, a strong classifier was designed by synthesizing the weak classifiers. The classifier with fewer samples was less robust while performing the classification.

Zemblys et al., 2019 implemented event detection using gazeNet without any requirement for hand-crafted signal features or signal thresholding. End-to-end deep learning was used in gazeNet, which categorized raw eye-tracking data into fixations, post-saccadic oscillations, and saccades. The problems created by unbalanced inputs were overcome by using heuristic data augmentation. However, the effect of previously classified information was required to be eliminated in gazeNet for enhancing event detection.

Friedman et al., 2023 developed the classification of eye movement using human manual classification (HMC). In manual classification, higher inter-rater reliability was used because it was an important representation of an expressive standardized categorization. For evaluating the results acquired from automatic classification, inter-rater reliability was used, alongside the training of machine learning approaches to achieving better classification. The HMC was effective only when operated with less input data during eye movement detection.

Yuan et al., 2021 presented the multi-source information-embedded approach (MSIEA) to investigate driving actions. A precise eye gaze was estimated by using the identification of eye gaze without gaze calibration. The information of multiscale sparse features of eye and head poses was combined for predicting the direction of gaze. Next, fused data were obtained by integrating the estimated gaze with vehicle data. The FastICA was used for discovering a large amount of driving-related data which were used for understanding the driving actions. The driver’s head orientations affected the performances of the MSIEA.

Kanade et al., 2021 developed gaze classification using convolutional neural networks (CNNs) for vehicular environments. From the input, the images of the face, right eye, and left eye were acquired using region-of-interest. Appropriate gaze features were obtained by fine-tuning the VGG-face network with pre-trained CNNs. Here, the classification was performed using the distance factor. The learning methodologies used in eye-tracking were utilized for enhancing the performance of eye feature evaluation.

## 3 BILSTM–LICRSA method

**FIGURE 1:**
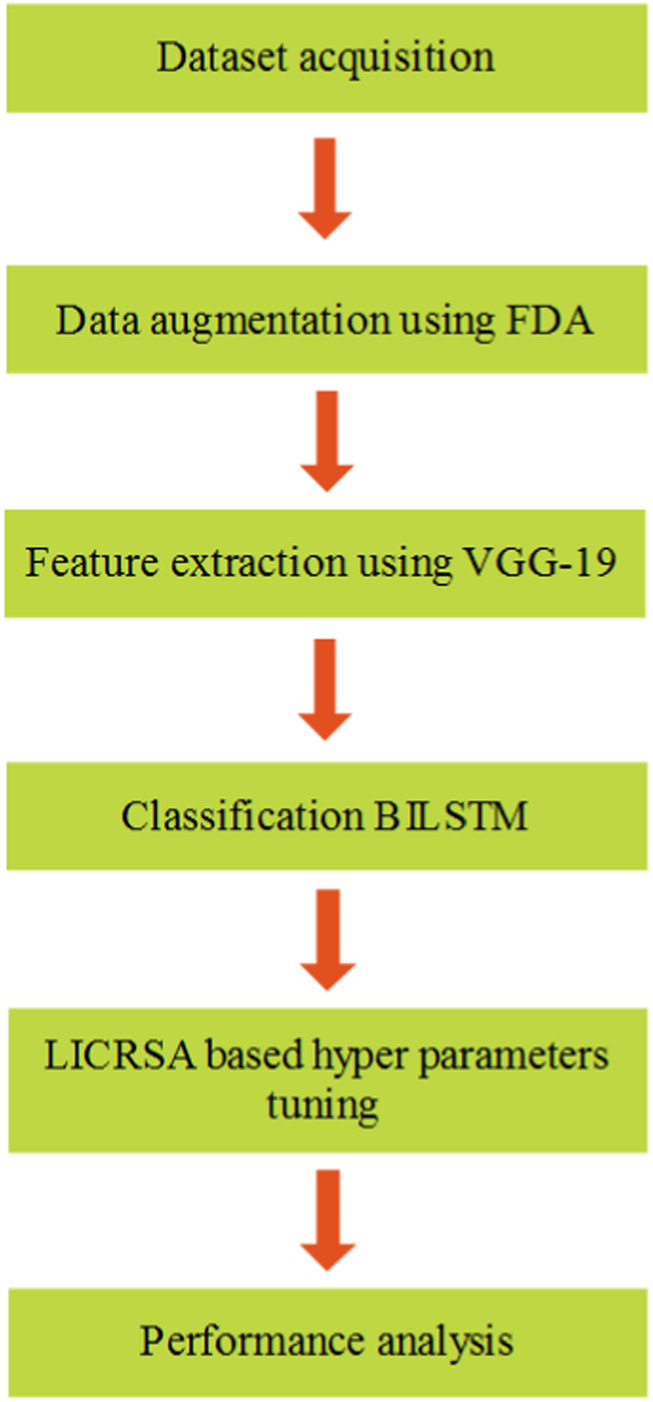
Block diagram of the BILSTM–LICRSA method.

The classification of eye movement event detection is performed using a BILSTM deep learning classifier, whereas the LICRSA is used to optimize the hyperparameters. The important processes of this proposed method are dataset acquisition, data augmentation, feature extraction using VGG-19, BILSTM classification, and LICRSA-based hyperparameter tuning process. The block diagram for the BILSTM–LICRSA method is shown in Figure 1.

### 3.1 Dataset acquisition

This research uses four different datasets: Lund2013 dataset (Larsson et al., 2013), collected dataset, GazeBaseR dataset (https://figshare.com/articles/dataset/GazeBase_Data_Repository/12912257.), and UTMultiView dataset (Sugano et al., 2014). Information about the datasets is given below:

#### 3.1.1 Lund2013

An annotated eye-tracker dataset, Lund2013 dataset, created at Humanities Lab (Larsson et al., 2013) was used to perform eye movement event detection. Monocular eye movement data of a person’s viewing images, videos, and moving dots are included in the Lund2013 dataset. The Lund2013 dataset has different classes such as fixations, saccades, smooth pursuit, post-saccadic oscillations, blinks, and undefined events. The data of fixations, saccades, and post-saccadic oscillations from the Lund2013 dataset are used in this research, which are 136,078 samples in total.

#### 3.1.2 Collected dataset

**FIGURE 2:**
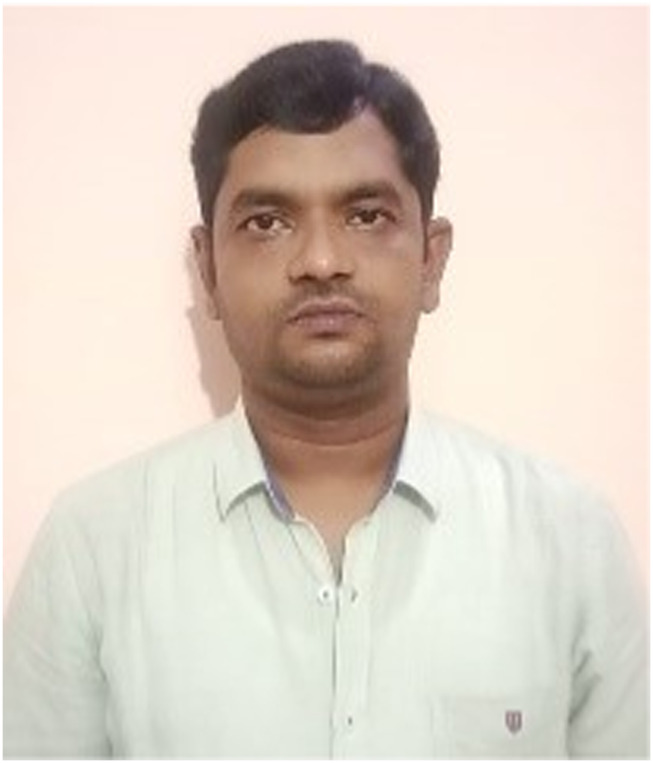
Sample real-time image.

Participants were made to sit directly facing the webcam at a fixed distance for collecting real-time face images. Videos are obtained by the webcam while the user follows a pre-defined on-screen target, i.e., a dot that moves in different locations. An onscreen target’s trajectory is recorded as the eye movement trajectory (EMT) in the system, and related angle images are saved using the real-time webcam. A sample real-time face image is shown in Figure 2. Both the images and EMT are combined as a collective dataset and used for real-time analysis. The total instances gathered are 10,000 from 100 test users, and it has the labels of fixations, saccades, and post-saccadic oscillations.

#### 3.1.3 GazeBaseR

The GazeBaseR dataset has temporal motion features of gaze points and spatial distribution features of saccades. The gaze point’s temporal motion features are a sequence of timestamps, and it is related to the gaze points, e.g., [0.1 s, (30, 20)], [0.2 s, (32, 22)], and [0.3 s, (31, 21)], which denotes the gaze point at various times. On the other hand, spatial distribution is a saccade vector series, e.g., (5, 3), (4, 2), and (3, 1), which denotes the distance and direction of eye movements. Two classes exist in this dataset during the prediction: movement or no movement.

#### 3.1.4 UTMultiView dataset

The UTMultiView has eye image and 3D head pose features, where the eye image is a grayscale and low-resolution image that has the iris, pupil, and some portion of the sclera. Next, the 3D head pose is a three-element vector (such as 30, 45, and 60) denoting a person’s head orientation by means of roll, pitch, and yaw. The prediction provides the three-element vector, e.g., 10, 20, and −10, denoting the evaluated direction of gaze.

The real-time images from the collected dataset and UTMultiView dataset are processed under FDA and VGG-19 to augment and extract features from the images. The text data are directly fed to the classifier along with the respective extracted features from VGG-19 for classification.

### 3.2 FDA-based data augmentation

FDA (Dabare et al., 2022) augments the collected data (i.e., images from the dataset), which is considered a preprocessing approach for avoiding overfitting issues in a classifier. There are two different phases that exist in the augmentation: fuzzification and generation of new augmented data. First, fuzzification is performed according to the clustering and identifying the membership grade of every record. A new input characteristic value is created according to the adequate cluster center value discovery and using the threshold value, which is an α -cut value. The parameters given as input to perform augmentation are specified in Table 1.

**TABLE 1:** Augmentation parameters.

Parameters	Description and value
Number of clusters	3
Bounding box $\left(X_{\min},Y_{\min},X_{\max},Y_{\max}\right)$	Pixel coordinates of the top-left and bottom-right corners of the face, e.g., $\left(100,50,200,150\right)$
Facial landmarks $\left(X,Y\right)$	Right eye center, e.g., $\left(170,70\right)$ , and left eye center, e.g., $\left(130,70\right)$
Head pose (roll, pitch, and yaw)	(roll—rotation around the X -axis, pitch—rotation around the Y -axis, yaw—rotation around the Z -axis), e.g., $\left(5,-10,15\right)$
Rotation (degrees)	Overall image, e.g., −15° to +15° (for subtle head motion)
Noise type	Gaussian noise: e.g., standard deviation $\left(\sigma \right)$ = 0.01 to 0.05—low-to-moderate noise; salt-and-pepper noise: e.g., noise density = 0.001 to 0.01—low-to-moderate noise

#### 3.2.1 Fuzzification of an entire input space

For the input data, the attributes are clustered using the fuzzy C-means (FCM) clustering method. The FCM method is selected because of the identification capacity for each cluster’s membership grade of every piece of information. The fuzzification process is described as follows:

1. FCM clusters the input data and deduces the membership degree of each attribute of the cluster. The usage of FCM clustering for unknown data offers a measure of belongingness, which is represented as the membership grade for every cluster. If unknown information is clustered, it belongs to various clusters. In FCM clustering, data have membership degrees either as 1 or 0 for the cluster center. The membership degree is 1, when the data belong to a respective cluster; otherwise, the membership degree is 0. Therefore, the values of membership degree are defined by the cluster.
2. The membership grade is considered in descending order for each attribute during the formation of the new membership dataset. For each record in the dataset, it is considered that the input contains n records and k attributes. Eqs 1, 2 show the input $\left(X\right)$ and output $\left(Y\right)$ spaces, respectively.

$$X=\left[\begin{array}{ccccc} x_{11} & x_{12} & x_{13} & \ldots & x_{1k}\\ x_{21} & x_{22} & x_{21} & \ldots & x_{2k}\\ x_{31} & x_{32} & x_{33} & \ldots & x_{3k}\\ \vdots & \vdots & \vdots & \ddots & \vdots \\ x_{n1} & x_{n2} & x_{n3} & \ldots & x_{nk} \end{array}\right].$$ (1)

$$Y={\left[y_{1}\;y_{2}\;y_{3}\;\ldots \;y_{n}\right]}^{T}.$$ (2)

Therefore, $y_{i}=x_{i1}\;x_{i2}\;x_{i3}\ldots x_{ik}$ , where the record of the dataset is represented as i . FCM clustering is performed over the input, and a new matrix $E_{i}$ is formed by the membership grade for each cluster as shown in Eq. 3.

$$E_{i}=\left[\begin{array}{c} \begin{array}{ccccc} \mu_{i1\left(X_{i1}\right)} & \mu_{i1\left(X_{i2}\right)} & \mu_{i1\left(X_{i3}\right)} & \ldots & \mu_{i1\left(X_{ik}\right)} \end{array}\\ \begin{array}{ccccc} \mu_{i2\left(X_{i1}\right)} & \mu_{i2\left(X_{i2}\right)} & \mu_{i2\left(X_{i3}\right)} & \ldots & \mu_{i2\left(X_{ik}\right)} \end{array}\\ \begin{array}{ccccc} \mu_{i3\left(X_{i1}\right)} & \mu_{i3\left(X_{i2}\right)} & \mu_{i3\left(X_{i3}\right)} & \ldots & \mu_{i3\left(X_{ik}\right)} \end{array} \end{array}\right],$$ (3)

where, μ represents the fuzzy membership grade for the dataset’s first record and $\mu_{i2}$ represents the second cluster. Eq 3 shows the transformation of input X into the element E , which is in descending order, as shown in Eq. 4.

$$E=\left[\begin{array}{c} \begin{array}{c} e_{1}\\ e_{2}\\ e_{3}\\ \vdots \end{array}\\ e_{n} \end{array}\right].$$ (4)

#### 3.2.2 New augmented dataset generation

**FIGURE 3:**
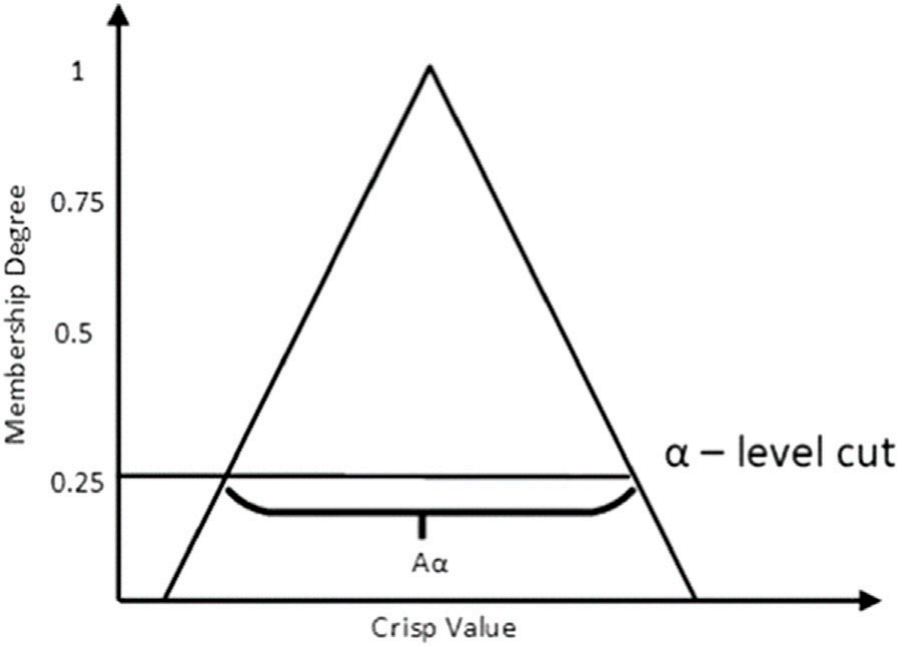
Sample of the triangular membership function for α -cut.

An allocation of cluster center α -cut for the fuzzified data is used to form the augmented data, wherein the steps are explained as follows:

1. Fuzzy uncertainty and fuzzy clustering are used to transform the input for identifying the appropriate cluster center of each data of cluster.
2. The fixed α -levels between 0 and 1 are horizontally cut by the membership function according to Figure 3, which illustrates a sample of triangular membership function at the α -cut level of 0.25. The membership function is horizontally cut between 0 and 1 with a restricted amount of α -levels. The uncertainty is high when there is a huge support for the membership function. The fuzzy set has all components in a membership function of $\alpha \left[0,1\right]$ , and the above is referred as the membership function’s α -cut. The finest augmented data are created by the optimum α -cut for generalization purposes without using an overall fuzzified data point. This FDA uses the trial-and-error approach to identify the optimum α -cut because an inadequate α -cut affects the balance of achieving the important features of gaze movements. According to the deliberation of how well the approach is generalized with the selected α -cut value, an optimum α -cut is selected for FDA. The group of elements that belong to the fuzzy set A and to the degree α is denoted as α -cut and is shown in Eq. 5, where the membership grade’s belongingness is denoted as $\mu_{A}$ .

$$A_{\alpha }=\left\{x\in X|\mu_{A}\left(x\right)\geq \alpha \right\}.$$ (5)

3. Data with a smaller membership degree than the selected α -cut are avoided from the new cluster center dataset.

Equation 6 is the identified threshold value for $E_{\alpha }$ , which represents the optimal α -cut value. The formulated threshold value is used for knowing the amount of data filtered from the augmented dataset. The threshold value, i.e., α -cut in $E_{\alpha }$ , is expressed in Eq. 6.

$$E_{\alpha }=\left\{u\in \left.U\right|\mu_{E}\left(u\right)\geq \alpha \right\},$$ (6)

where U denotes the universe of discourse, $\mu_{E}$ is the membership grade between $\left[0,1\right]$ , and value between $\left[0,1\right]$ is denoted as α . If the fuzzy α -cut is used in E , the number of rows in E is changed according to the fuzzy α -cut. Moreover, a cluster center dataset is formed by using the centers of each cluster. For instance, Eq. 7 shows the cluster center dataset for n records of original data and three clusters.

$$CL=\left[\begin{array}{ccc} {CL}_{11} & {CL}_{12} & {CL}_{13}\\ {CL}_{21} & {CL}_{22} & {CL}_{23}\\ \vdots & \vdots & \vdots \\ {CL}_{n1} & {CL}_{n2} & {CL}_{n3} \end{array}\right].$$ (7)

Hence, the cluster center of each element is transformed as the CL cluster center dataset. The cluster center is considered a symbol of fuzzy values of data. Subsequently, the identified cluster centers are included to the input dataset to generate the augmented dataset, as expressed in Eq. 8.

$$Aug\;data=\{X,\left.CL\right\}.$$ (8)

**FIGURE 4:**
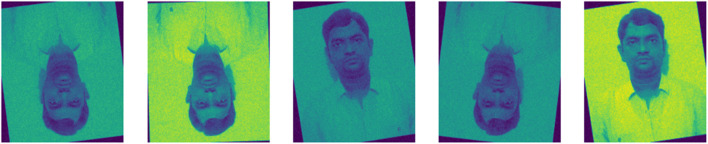
Sample augmented image.

The augmented data from the FDA is concatenated with the input data results, in 305,744 samples, which is 55.49% is higher than the given input. The sample for the augmented real-time image used along with the input is shown in Figure 4.

### 3.3 Feature extraction using VGG-19

In feature extraction, the VGG-19 (Mateen et al., 2019) is used to obtain important features from the augmented input $\left(Aug\;data\right)$ , i.e., augmented images. Generally, VGG-19 is a deep neural network with multilayered operation. Due to its simple architecture, VGG-19 is suitable, and the 3×3 convolutional layers are positioned at the top to increase the depth level. In VGG-19, the max-pooling layers are used as a handler to minimize volume size, and the two fully connected layers are used with 4,096 neurons. Here, feature extraction is accomplished by convolutional layers, and the dimensionality of features is reduced by max-pooling layers related to the convolutional layers. In the first convolutional layer, 64 kernels are used to accomplish the feature extraction, followed by a feature vector generated by fully connected layers. For each sample, VGG-19 returns 4,096 features during feature extraction. The extracted feature vectors are given as input for BILSTM classification.

### 3.4 Classification using BILSTM

**FIGURE 5:**
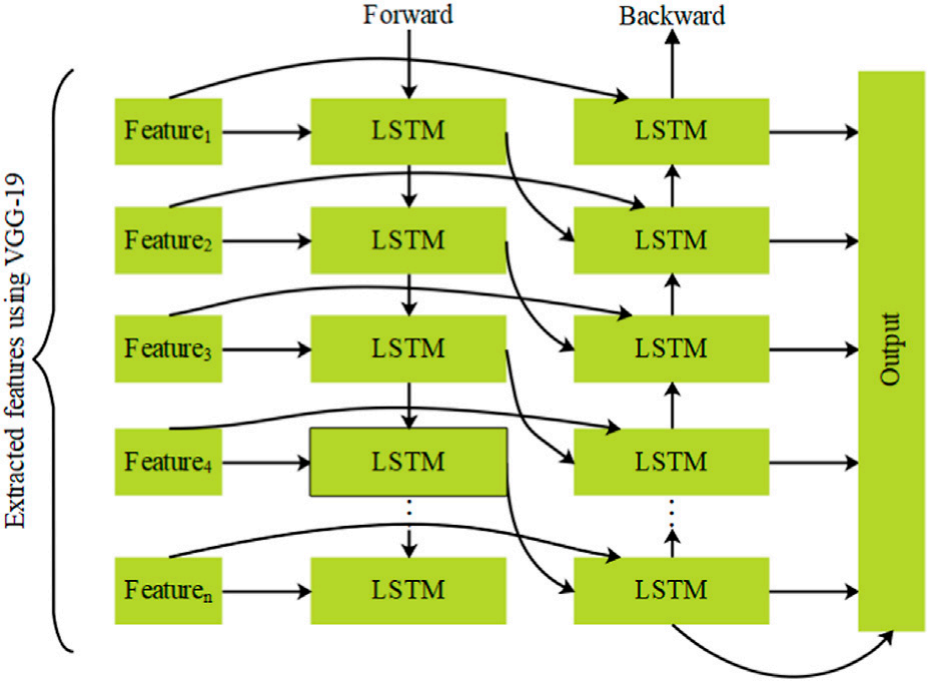
Architecture of BILSTM.

The features from VGG-19 are given along with the text features from the respective dataset as input to the BILSTM (Ali et al., 2021) for classifying eye movements. In general, LSTM is an extended version of a recurrent neural network with a similar kind of architecture. The RNN and LSTM transfer the data from one stage to another stage. The LSTM classification offers higher success in longstanding dependencies. However, a single LSTM unit is restricted to classifying the output according to the previous data. Hence, the single LSTM has the possibility of providing misclassification without considering the forward data. Accordingly, the BILSTM approach is developed, which incorporates both the past and upcoming data, therefore enhancing the classification. There are two LSTM models operated in a parallel manner, as shown in Figure 5. In that, one LSTM operates from the input data’s start, and the other operates from the input data’s end. Consequently, the BILSTM classification supports both the previous and upcoming data. For instance, the first and second LSTM of BILSTM studies the data starting from left to right and ending from right to left. This helps the BILSTM model completely keep the information of eye movement data for classification.

The BILSTM model shown in Figure 5 is used in the hidden layers, which have the capacity for keeping older data for a short time. An essential element in the BILSTM model is a memory cell $C_{t}$ that is updated using the input gate $\left(i_{t}\right)$ and forget gate $\left(f_{t}\right)$ . The data required to be kept in the memory cell are decided by using the input gate. On the other hand, the data required to be dumped from the memory cell are decided by the forget gate. The $C_{t}$ of forward LSTM in every time step is updated by using Eqs 9–14.

$$u_{t}^{\left(f\right)}=\tanh\left(w_{xu}^{\left(f\right)}x_{t}+w_{hu}^{\left(f\right)}h_{t-1}+b_{u}^{\left(f\right)}\right).$$ (9)

$$i_{t}^{\left(f\right)}=\sigma \left(w_{xi}^{\left(f\right)}x_{t}+w_{hi}^{\left(f\right)}h_{t-1}+b_{i}^{\left(f\right)}\right).$$ (10)

$$f_{t}^{\left(f\right)}=\sigma \left(w_{xf}^{\left(f\right)}x_{t}+w_{hf}^{\left(f\right)}h_{t-1}+b_{f}^{\left(f\right)}\right).$$ (11)

$$C_{t}^{\left(f\right)}=f_{t}^{\left(f\right)}⊙C_{t-1}^{\left(f\right)}+i_{t}^{\left(f\right)}⊙u_{t}^{\left(f\right)}.$$ (12)

$$O_{t}^{\left(f\right)}=\sigma \left(w_{xo}^{\left(f\right)}x_{t}+w_{ho}^{\left(f\right)}h_{t-1}+b_{o}^{\left(f\right)}\right).$$ (13)

$$fh_{t}=O_{t}^{\left(f\right)}⊙{\tanh(C}_{t}^{\left(f\right)}).$$ (14)

The $C_{t}$ of backward LSTM in every time step is updated by using Eqs 15–20.

$$u_{t}^{\left(b\right)}=\tanh\left(w_{xu}^{\left(b\right)}x_{t}+w_{hu}^{\left(b\right)}h_{t+1}+b_{u}^{\left(b\right)}\right).$$ (15)

$$i_{t}^{\left(b\right)}=\sigma \left(w_{xi}^{\left(b\right)}x_{t}+w_{hi}^{\left(b\right)}h_{t+1}+b_{i}^{\left(b\right)}\right).$$ (16)

$$f_{t}^{\left(b\right)}=\sigma \left(w_{xf}^{\left(b\right)}x_{t}+w_{hf}^{\left(b\right)}h_{t+1}+b_{f}^{\left(b\right)}\right).$$ (17)

$$C_{t}^{\left(b\right)}=f_{t}^{\left(b\right)}⊙C_{t-1}^{\left(b\right)}+i_{t}^{\left(b\right)}⊙u_{t}^{\left(b\right)}.$$ (18)

$$O_{t}^{\left(b\right)}=\sigma \left(w_{xo}^{\left(b\right)}x_{t}+w_{ho}^{\left(b\right)}h_{t+1}+b_{o}^{\left(b\right)}\right).$$ (19)

$$bh_{t}=O_{t}^{\left(b\right)}⊙{\tanh(C}_{t}^{\left(b\right)}).$$ (20)

Here, the parameters that need to be learned in the BILSTM classification are $w_{xi},b_{i},w_{xu},w_{hu},w_{xo},b_{o},w_{xf,}$ and $b_{f}$ , and the input of BILSTM is $x_{t}$ . The forward and backward LSTM outputs are denoted as ${fh}_{t}$ and ${bh}_{t}$ , respectively. BILSTM has the capacity to read data in both the directions, i.e., forward and backward. In forward LSTM, data are processed from left to right, while data are processed from right to left in backward LSTM. The combination of forward and backward LSTM outputs is the outcome of BILSTM $\left(H_{T}\right)$ for each time step t that is expressed in Eq. 21.

$$H_{T}=w_{x}^{\left(h\right)}fh_{t}+w_{h}^{\left(h\right)}bh_{t}+b^{\left(h\right)},$$ (21)

where BILSTM has $fh_{t}$ and $bh_{t}$ that denote the past and future data, respectively. Therefore, the BILSTM approach combines the past and future backgrounds and considers the BILSTM’s output.

### 3.5 LICRSA-based hyperparameter tuning for BILSTM

The important goal of this work is to optimize BILSTM’s hyperparameters using the LICRSA (Huang et al., 2022) and obtain improved performance in the classification. In general, the RSA replicates the predation plan and social activities of crocodiles. Hyperparameters such as dropout, learning rate, L2 regularization, and max-epoch are optimized using the LICRSA. The LICRSA starts from the initial solutions, i.e., randomly initializes the hyperparameters and helps enhance the classification. The fitness function considered for BILSTM is to perform the analysis and return the accuracy of eye movement classification.

#### 3.5.1 An iterative process of the LICRSA for hyperparameter tuning

The solutions of the LICRSA are initialized using the minimum and maximum values of dropout, learning rate, L2 regularization, and max-epoch. The range of dropout is $\left[0.1,0.4\right]$ , that of learning rate is $\left[0.003,0.1\right]$ , and that of L2 regularization is $\left[0.003,0.1\right]$ , whereas the max-epoch has choices of $\left[5,10,15,20\right]$ . This randomly generated solution is given as input to the LICRSA for finding the optimal set of hyperparameters. Lévy flight and interaction crossover are selected for enhancing the search process. The Lévy flight approach is used to search local solutions and improve the precision, whereas the algorithm development is improved by interaction crossover. In this study, the Lévy flight approach was used for creating a random number with replacement under the following features: 1) the created random values are huge sometimes; however, they are mostly interspersed with small values in between, and 2) a step’s probability density function is heavy-tailed. The generated random number is used to perform location updates for generating oscillations and accomplish small foraging in the neighborhood based on fluctuations of the random value in one round and further, thus further helping the candidate solution come out of the local optimum.

Equation 22 expresses the definition of Lévy dissemination.

$$Levy\left(\gamma \right)∼u=l^{-1-\gamma },0<\gamma \leq 2,$$ (22)

where u signifies the Gaussian distribution and the iteration is denoted as l . In the encircling process, the Lévy flight is used for high and belly walking movements of crocodiles to increase the searching area. Moreover, the optimal exploitation phase’s flexibility is improved by using the Lévy flight in hunting coordination and cooperation. The encircling is performed according to the Lévy flight expressed in Eq. 23.

$$y_{\left(i,j\right)}\left(l+1\right)=\left\{\begin{array}{c} y_{j}^{*}\;\left(l\right)\times -\varphi_{\left(i,j\right)}\left(l\right)\times \mu -{RF}_{\left(i,j\right)}\left(l\right)\times \lambda \times Levy\left(\gamma \right),l\leq \frac{T_{\max}}{4}\\ y_{j}^{*}\;\left(l\right)\times y_{\left(r_{1},j\right)}\times ES\left(l\right)\times \lambda \times Levy\left(\gamma \right),\frac{T_{\max}}{4}\leq l<\;\frac{{2\times T}_{\max}}{4} \end{array}\right.,$$ (23)

where the location j of solution i is denoted as $y_{\left(i,j\right)}$ , $y_{j}^{*}\;\left(l\right)$ denotes the best solution, $\varphi_{\left(i,j\right)}\left(l\right)$ is the operator of the crocodile i in dimension j,μ is fixed as 0.1, which is used to handle the search accuracy, the reduced function is denoted as ${RF}_{\left(i,j\right)},\lambda$ is fixed as 0.1, the stochastic integer in the range of $\left[1,N\right]$ is denoted $r_{1},N$ is the number of solutions in LICRSA, $T_{\max}$ denotes the maximum iterations, and the evolutionary sense is $ES\left(l\right)$ . Eq. 24 is the LICRSA’s hunting activities based on the Lévy flight.

$$y_{\left(i,j\right)}\left(t+1\right)=\left\{\begin{array}{l} y_{j}^{*}\;\left(l\right)\times P_{\left(i,j\right)}\left(l\right)\times \lambda \times Levy\left(\gamma \right),\frac{{2\times T}_{\max}}{4}\leq l<\frac{{3\times T}_{\max}}{4}\\ y_{j}^{*}\;\left(l\right)-\varphi_{\left(i,j\right)}\left(t\right)\times \epsilon -{RF}_{\left(i,j\right)}\left(t\right)\times \lambda \times Levy\left(\gamma \right),\frac{{3\times T}_{\max}}{4}\leq l<T_{\max} \end{array}\right.,$$ (24)

where a difference in the percentage among the crocodiles in the best location and current location is denoted as $P_{\left(i,j\right)}$ , and ϵ is a small value.

The candidate solutions in the current location are readjusted using interaction crossover based on the information exchange of the candidate in the best solution and two candidate solutions. The new location obtains data over optimal and remaining candidate solutions for enhancing the ability to search for achieving an optimal set of hyperparameters. Initially, the parameter CF expressed in Eq. 25 is used to handle the crocodile population’s activity, which linearly decreases along with iterations.

$$CF={\left(1-\frac{l}{T_{\max}}\right)}^{2\times \frac{l}{T_{\max}}}.$$ (25)

The population in the LICRSA is randomly separated into two portions with the same amount of crocodiles. The selected portions are $y_{k1}$ and $y_{k2}$ , and these locations are communicated for updating two crocodiles. Eqs 26, 27 show the updated strategy of the LICRSA.

$$y_{\left(k1,j\right)}\left(l+1\right)=y_{\left(k1,j\right)}\left(l\right)+CF\times \left(y_{j}^{*}\;\left(l\right)-y_{\left(k1,j\right)}\left(l\right)\right)+c_{1}\left(y_{\left(k1,j\right)}\left(l\right)-y_{\left(k2,j\right)}\left(l\right)\right),$$ (26)

$$y_{\left(k2,j\right)}\left(l+1\right)=y_{\left(k2,j\right)}\left(l\right)+CF\times \left(y_{j}^{*}\;\left(l\right)-y_{\left(k2,j\right)}\left(l\right)\right)+c_{2}\left(y_{\left(k2,j\right)}\left(l\right)-y_{\left(k1,j\right)}\left(l\right)\right),$$ (27)

where stochastic integers in the range of $\left[0,1\right]$ are $c_{1}$ and $c_{2}$ and the crocodile in location k1 is $y_{k1}$ . After performing interactive crossover, the crocodiles with lesser capacities are eliminated using the elimination mechanism, as shown in Eq. 28.

$$y_{\left(i,j\right)}\left(l+1\right)=\left\{\begin{array}{l} y_{\left(i,j\right)}\left(l\right)\;if\;f\left(y_{\left(i,j\right)}\left(l\right)\right)<f\left(y_{\left(i,j\right)}\left(l+1\right)\right)\\ y_{\left(i,j\right)}\left(l+1\right),elseif\;f\left(y_{\left(i,j\right)}\left(l+1\right)\right)<f\left(y_{\left(i,j\right)}\left(l\right)\right)\; \end{array}\right..$$ (28)

The evaluation measures considered for this research are accuracy, precision, sensitivity, and F1-score, which are expressed in Eqs 29–32.

$$Accuracy=\frac{TP+TN}{TN+TP+FN+FP}\times 100,$$ (29)

$$Precision=\frac{TP}{TP+FP}\times 100,$$ (30)

$$Sensitivity=\frac{TP}{TP+FN}\times 100,$$ (31)

$$F1-score=2\times \frac{Sensitivity\times Precision}{Sensitivity+Precision}\times 100,$$ (32)

where TP is true positive, TN is true negative, FP is false positive, and FN is false negative. Furthermore, the measure of the AUROC is used to know how well the model differentiates among the classes according to the true-positive rate versus the false-positive rate. Moreover, the AUPRC computes an amount of true positives divided by the addition of true positives and false positives. These AUROC and AUPRC are computed for multi-class classification via a macro averaging process. In macro averaging, AUROC and AUPRC are individually computed for each class, and the average value is taken among all classes.

## 4 Results and discussion

The proposed eye movement detection is implemented and simulated using MATLAB R 2020a software, where the system functions with an i5 processor and 8 GB RAM. The proposed method is used to classify the eye movements of disabled people.

### 4.1 Performance analysis

Eye movement event detection is an important objective of this proposed method, which is specifically designed for disabled people. For performing eye movement event detection and classification, data from the Lund2013 and collected datasets acquired in real-time are used for analysis. However, event detection using eye movement is not implemented by many types of research; hence, two more datasets: GazeBaseR dataset (https://figshare.com/articles/dataset/GazeBase_Data_Repository/12912257.) and UTMultiView (Sugano et al., 2014) are considered for further analyzing the proposed method. A total of 322 subjects are included in the GazeBaseR dataset, where everyone has completed two recording sessions. Moreover, UTMultiView has 24,320 samples, which include head pose and gaze directions. The training and testing ratio of 70:30 is considered for evaluating the BILSTM–LICRSA. In this section, the proposed method is evaluated with different classifiers and optimization algorithms for the hyperparameter tuning process.

#### 4.1.1 BILSTM–LICRSA evaluation with different classifiers

**FIGURE 6:**
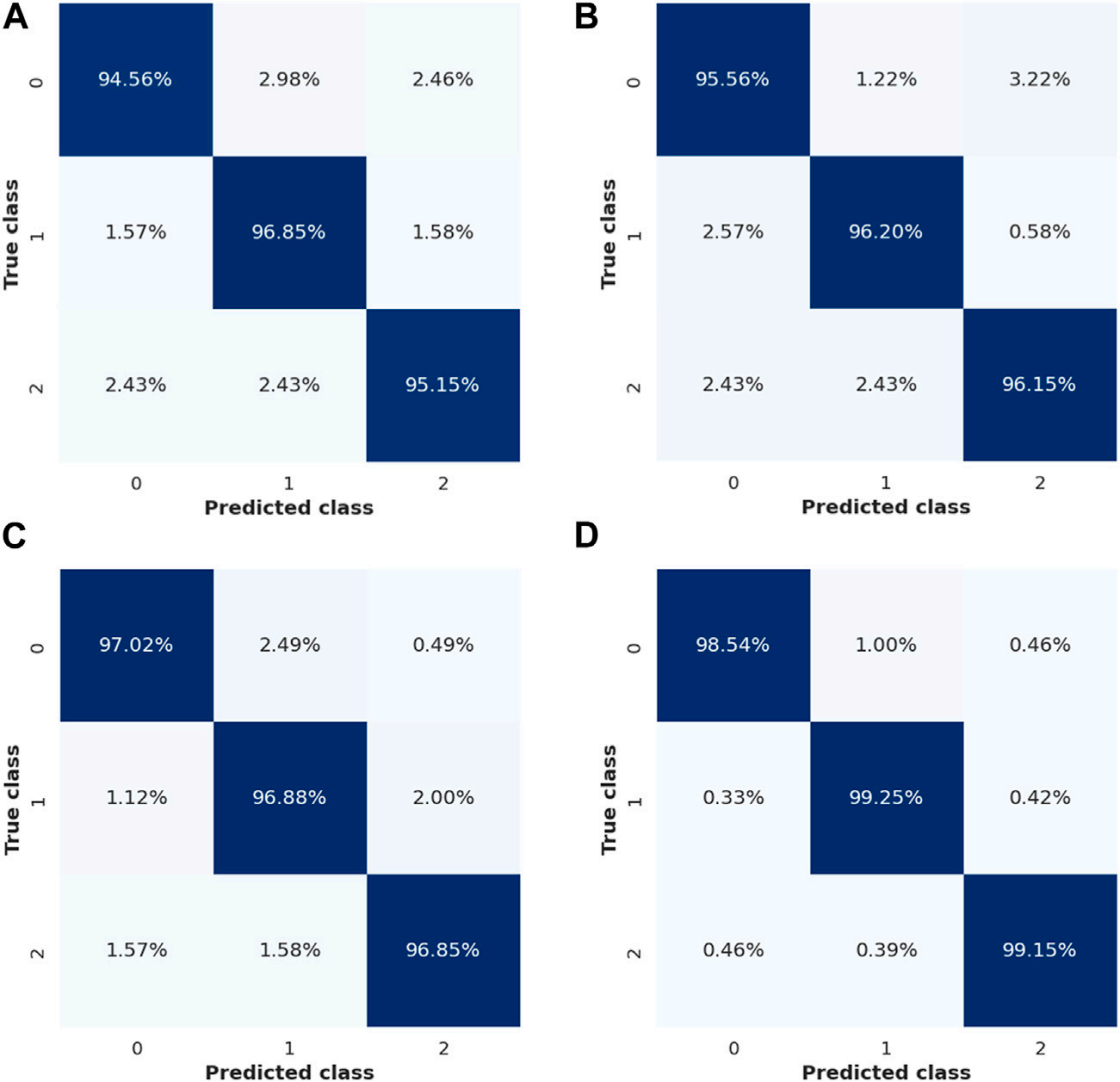
Confusion matrix. **(A)** GAN, **(B)** RNN, **(C)** LSTM, and **(D)** BILSTM.

This section shows the performance of BILSTM with different classifiers such as GAN, RNN, and LSTM. The confusion matrix (CM) of the GAN, RNN, LSTM, and BILSTM for the Lund2013 dataset is shown in Figure 6. The numbers 0, 1, and 2 represent the classes of fixations, saccades, and post-saccadic oscillations, respectively. This CM is used to determine how well the developed model performs an effective classification. From the analysis, it is concluded that BILSTM offers a better performance than the GAN, RNN, and LSTM.

Here, the LICRSA-based hyperparameter tuning is incorporated into the classifiers. The analysis of BILSTM with different classifiers is shown in Tables 2–5 for Lund2013, collected, GazeBaseR, and UTMultiView datasets, respectively. From these analyses, it is found that the proposed BILSTM method provides better performance than the GAN, RNN, and LSTM. For example, the accuracy of BILSTM for the GazeBaseR dataset is 98.95%, whereas the GAN obtains 95.93%, RNN obtains 96.80%, and LSTM obtains 97.40%. The reasons for BILSTM having superior performances are stated as follows: 1) the combined information of both the past and upcoming data is used to avoid misclassification while classifying the data and 2) the hyperparameter tuning process developed for BILSTM further enhances the classification.

**TABLE 2:** BILSTM–LICRSA evaluation with different classifiers for the Lund2013 dataset.

Classifiers	Accuracy (%)	Sensitivity (%)	Precision (%)	F1-score (%)	AUROC	AUPRC
GAN	95.65	94.68	94.11	95.73	0.914	0.927
RNN	96.28	97.50	96.27	96.81	0.930	0.942
LSTM	97.57	98.56	98.78	97.42	0.951	0.962
BILSTM	99.32	99.86	99.80	99.05	0.971	0.983

**TABLE 3:** BILSTM–LICRSA evaluation with different classifiers for the collected dataset.

Classifiers	Accuracy (%)	Sensitivity (%)	Precision (%)	F1-score (%)	AUROC	AUPRC
GAN	92.44	92.88	93.64	93.25	0.908	0.914
RNN	94.81	94.70	94.88	94.78	0.911	0.922
LSTM	96.10	96.06	95.05	95.55	0.937	0.940
BILSTM	97.64	96.58	97.15	96.86	0.954	0.962

**TABLE 4:** BILSTM–LICRSA evaluation with different classifiers for the GazeBaseR dataset.

Classifiers	Accuracy (%)	Sensitivity (%)	Precision (%)	F1-score (%)	AUROC	AUPRC
GAN	95.93	95.18	94.39	95.61	0.928	0.939
RNN	96.80	95.30	95.81	96.88	0.931	0.944
LSTM	97.40	96.39	96.71	97.88	0.942	0.956
BILSTM	98.95	99.37	98.76	98.99	0.964	0.971

**TABLE 5:** BILSTM–LICRSA evaluation with different classifiers for the UTMultiView dataset.

Classifiers	Accuracy (%)	Sensitivity (%)	Precision (%)	F1-score (%)	AUROC	AUPRC
GAN	95.31	95.49	94.15	95.04	0.919	0.923
RNN	96.49	96.08	97.04	97.06	0.930	0.942
LSTM	98.15	98.35	98.27	98.02	0.951	0.960
BILSTM	98.96	99.34	99.22	98.91	0.966	0.971

The performance evaluation for the BILSTM–LICRSA according to the augmentation is shown in Table 6. This shows that BILSTM–LICRSA with augmented data from the FDA improves the classification than the actual input. The BILSTM–LICRSA with FDA achieves enhanced performance compared to the classifier with actual input by avoiding the overfitting issue.

**TABLE 6:** Analysis of accuracy based on FDA.

Dataset	Accuracy (%)	
	Actual input	Actual with augmented input from FDA
Lund2013	95.11	99.32
Collected dataset	93.57	97.64
GazeBaseR	94.57	98.95
UTMultiView	95.81	98.96

The bootstrapping average precision (AP) and rank for different classifiers is shown in Table 7. According to the statistics, an average precision is investigated from a single test set sample, that is, the point evaluation. The point evaluation is varied with the usage of various test sets for investigation, which falls into confidence intervals with a definite probability. These confidence intervals are utilized for evaluating the difference among the algorithms. This analysis represents that BILSTM has the first grade among other classifiers.

**TABLE 7:** Analysis of bootstrapping AP and rank.

Classifiers	AP range			Rank
	0.025	0.5	0.975	
GAN	93.19	94.21	95.64	4
RNN	94.55	94.80	96.79	3
LSTM	95.17	96.04	97.98	2
BILSTM	95.68	97.58	99.77	1

#### 4.1.2 BILSTM–LICRSA evaluation with different optimizations

This section shows the performance of the LICRSA with different optimization algorithms such as PSO, GWO, and RSA. The evaluation of BILSTM–LICRSA with different optimization algorithms for Lund2013, collected, GazeBaseR, and UTMultiView datasets is given in Tables 8–11, respectively. This analysis shows that the LICRSA achieves improved classification than PSO, GWO, and RSA. The LICRSA with BILSTM for the GazeBaseR dataset achieves an accuracy of 98.95%, whereas the PSO obtains 93.56%, GWO obtains 96.24%, and RSA obtains 97.89%. In LICRSA, the Lévy flight and interactive crossover are used for searching the local solutions and enhancing the ability to search, which is further used to achieve the optimal set of hyperparameters. This helps improve the classification of eye movement.

**TABLE 8:** BILSTM–LICRSA evaluation with different optimization algorithms for the Lund2013 dataset.

Optimization algorithms	Accuracy (%)	Sensitivity (%)	Precision (%)	F1-score (%)	AUROC	AUPRC
PSO	94.14	94.07	95.84	95.58	0.930	0.939
GWO	96.39	97.73	96.47	96.53	0.942	0.951
RSA	97.87	98.46	97.74	97.93	0.957	0.964
LICRSA	99.32	99.86	99.80	99.05	0.971	0.983

**TABLE 9:** BILSTM–LICRSA evaluation with different optimization algorithms for the collected dataset.

Optimization algorithms	Accuracy (%)	Sensitivity (%)	Precision (%)	F1-score (%)	AUROC	AUPRC
PSO	93.81	93.84	94.05	93.94	0.912	0.921
GWO	95.28	95.04	95.79	95.41	0.927	0.949
RSA	96.50	95.68	96.45	96.06	0.942	0.953
LICRSA	97.64	96.58	97.15	96.86	0.954	0.962

**TABLE 10:** BILSTM–LICRSA evaluation with different optimization algorithms for the GazeBaseR dataset.

Optimization algorithms	Accuracy (%)	Sensitivity (%)	Precision (%)	F1-score (%)	AUROC	AUPRC
PSO	93.56	94.62	93.80	93.24	0.925	0.930
GWO	96.24	95.61	95.93	95.72	0.931	0.949
RSA	97.89	96.87	96.88	96.68	0.950	0.953
LICRSA	98.95	99.37	98.76	98.99	0.964	0.971

**TABLE 11:** BILSTM–LICRSA evaluation with different optimization algorithms the UTMultiView dataset.

Optimization algorithms	Accuracy (%)	Sensitivity (%)	Precision (%)	F1-score (%)	AUROC	AUPRC
PSO	94.35	94.53	93.66	94.51	0.929	0.921
GWO	95.49	95.95	96.53	95.74	0.937	0.941
RSA	98.40	97.26	97.96	97.18	0.954	0.969
LICRSA	98.96	99.34	99.22	98.91	0.966	0.971

### 4.2 Comparative analysis

The comparative analysis of BILSTM–LICRSA with existing methods such as the gazeNet (Zemblys et al., 2019), HMC (Friedman et al., 2023), and MSIEA (Yuan et al., 2021) is provided in this section. The comparative analysis is provided for three different datasets: Lund2013, GazeBaseR, and UTMultiView. Here, the gazeNet (Zemblys et al., 2019), HMC (Friedman et al., 2023), and MSIEA (Yuan et al., 2021) are considered for comparisons of the Lund2013 dataset, GazeBaseR dataset, and UTMultiView dataset, respectively. The evaluation of BILSTM–LICRSA with gazeNet (Zemblys et al., 2019), HMC (Friedman et al., 2023), and MSIEA (Yuan et al., 2021) is shown in Tables 12–14. This comparison denotes that the BILSTM–LICRSA accomplishes improved classification than the gazeNet (Zemblys et al., 2019), HMC (Friedman et al., 2023), and MSIEA (Yuan et al., 2021). The LICRSA is used to identify the optimal set of hyperparameters, alongside the utilization of past and upcoming data in BILSTM being utilized to enhance the classification.

**TABLE 12:** Comparison for the Lund2013 dataset.

Methods	Accuracy (%)	F1-score (%)
gazeNet Zemblys et al. (2019)	88.97	98
BILSTM–LICRSA	99.32	99.05

**TABLE 13:** Comparison for the GazeBaseR dataset.

Methods	F1-score (%)
HMC (Friedman et al., 2023)	93.46
BILSTM–LICRSA	98.99

**TABLE 14:** Comparison for the UTMultiView dataset.

Methods	F1-score (%)
MSIEA (Yuan et al., 2021)	52.9
BILSTM–LICRSA	98.91

### 4.3 Discussion

This section offers a detailed discussion related to the outcomes of BILSTM–LICRSA developed for eye movement event classification. Initially, the results of BILSTM were investigated with different state-of-the-art classifiers such as the GAN, RNN, and LSTM. Next, different optimization algorithms such as PSO, GWO, and RSA were used to investigate the efficiency of optimal hyperparameters discovered from the LICRSA. The developed BILSTM–LICRSA method is analyzed with four datasets: Lund2013, collected dataset, GazeBaseR, and UTMultiView. The evaluation of results represents that the BILSTM–LICRSA achieves a better performance than the GAN, RNN, LSTM, PSO, GWO, and RSA. Moreover, the BILSTM–LICRSA has better performance than the existing gazeNet (Zemblys et al., 2019), HMC (Friedman et al., 2023), and MSIEA (Yuan et al., 2021). BILSTM presents robust classification by integrating both the past and upcoming data during the recognition. Moreover, the optimum hyperparameters obtained from the LICRSA additionally help improve the classification.

## 5 Conclusion

In this paper, an effective eye movement event classification is achieved by using BILSTM with a hyperparameter tuning process. The LICRSA-based hyperparameter tuning is done according to the accuracy for improving the classification process. The Lévy flight and interactive crossover are used for searching the local solutions and improving the searching ability to achieve the optimal set of hyperparameters. On the other hand, the utilization of past and upcoming data in BILSTM further enhances the classification. The issue related to overfitting is avoided by using FDA-based augmentation. Therefore, the combination of BILSTM and the LICRSA achieves better classification of eye movements. The outcomes of the BILSTM–LICRSA show that it outperforms the gazeNet, HMC, and MSIEA. The F1-score of BILSTM–LICRSA for the GazeBaseR dataset is 98.99%, which is superior to that of the MSIEA. In future, different ways of feature aggregation can be studied to enhance the performance of the proposed eye movement event classification.

## Data Availability

The original contributions presented in the study are included in the article/Supplementary Material; further inquiries can be directed to the corresponding author.
